# Relating genes in the biosynthesis of the polyphenol composition of Andean colored potato collection

**DOI:** 10.1002/fsn3.69

**Published:** 2013-12-18

**Authors:** Leslie Tejeda, Juan Antonio Alvarado, Magdalena Dębiec, José Mauricio Peñarrieta, Oscar Cárdenas, Maria Teresa Alvarez, Aakash Chawade, Lars Nilsson, Björn Bergenståhl

**Affiliations:** 1 School of Chemistry Faculty of Pure and Natural Sciences, San Andrés UniversityP.O. Box 303, La Paz, Bolivia; 2 Food Colloids Group Department of Food Technology, Engineering and Nutrition, Lund UniversityP.O. Box 124, S-221 00, Lund, Sweden; 3 Drug Research Institute and Biochemical Faculty of Pharmaceutical and Biochemical Sciences, San Andrés UniversityP.O. Box 303, La Paz, Bolivia; 4 Department of Immunotechnology, Lund UniversityBMC D13, SE-22184, Lund, Sweden

**Keywords:** *Ans* and *stan* genes, anthocyanidins, antioxidant capacity, polyphenolic compounds and purple potatoes

## Abstract

The objective of this study was to evaluate total antioxidant capacity (TAC), total phenolic content (TPH), and the identification of anthocyanidin and polyphenolic compounds in 13 colored potatoes collected from the Andean region of Bolivia, and understand how the chemical composition correlated with the botanical classification and molecular characterization of genes, *ans* (anthocyanidin synthase) and *stan1* (*Solanum tuberosum* anthocyanidin synthase), associated with the synthesis of anthocyanidins. The results show the existence of a limited correlation between botanical classification, based on morphological identification and polyphenol composition. No association between genetic grouping of the *ans* and *stan* genes and botanical classification was found. However, it was possible to identify a correlation between the *ans* gene clades and the levels of anthocyanidins as well as of other polyphenols. Thus, this result confirms the concept that potato color can be used in the search for high polyphenol potato cultivars.

## Introduction

Potatoes accumulate a great variety of secondary metabolites, including polyphenolic and many other phytochemical compounds, as a protection against the adverse effects of solar radiation, and injury of herbivorous insects or pathogens such as bacteria or fungi (Del Mar Verde Méndez et al. 2004; Brown 2005). Polyphenolic compounds are able to form stable radicals and thereby act as radical scavengers and antioxidants. As antioxidants, polyphenols are expected to have potentially positive health benefits (Del Mar Verde Méndez et al. 2004). Anthocyanidins is a group of polyphenols able to create red, purple, blue, or orange colors (Brown 2005).

Colored tubers contained mostly pelargonidin, peonidin, petunidin, malvidin, cyanidin, and delphinidin (Lewis et al. 1998; Alcalde-Eon et al. 2004; Hamouz et al. 2011; Lachman et al. 2012). Tuber parenchyma tissue also contained chlorogenic acid and other phenolic acids, plus low concentration of flavonoids. Determinations of polyphenols in periderm tissue have shown higher level of chlorogenic acid, moderate amounts of protocatechuic acid, caffeic acid, vanillic acid, and sinapic acid, and low concentrations of gallic acid, syringic acid, catechin, p-coumaric acid, ferulic acid, salicylic acid, and cinnamic acid, where chlorogenic acid constitutes ∼90% of the total phenolic content (Friedman 1997; Lewis et al. 1998; Del Mar Verde Méndez et al. 2004; Preedy et al. 2011).

**Figure 1:**
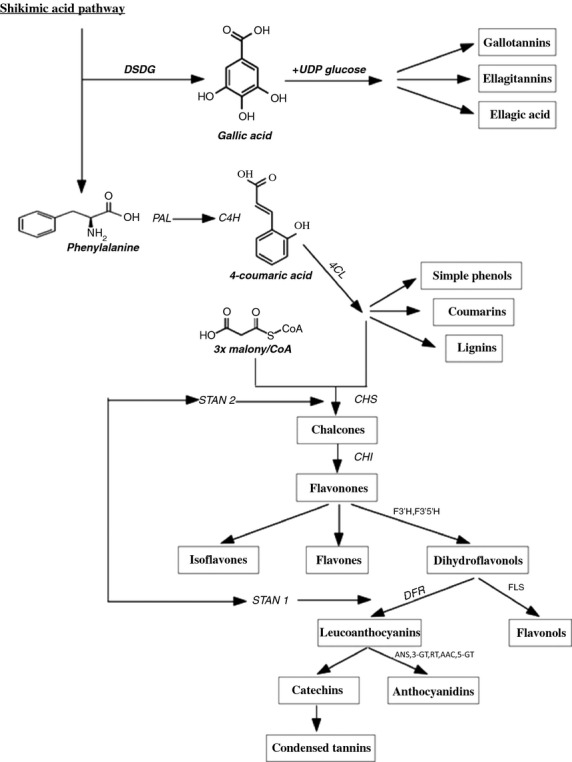
Biosynthesis of phenolic compounds (modified after Stushnoff et al. 2010; Al-Sane et al. 2011; Bai et al. 2011; Lachman et al. 2012). ANS, anthocyanidin synthase; CHI, chalconeisomerase; CHS, chalconesynthetase; C4H, cinnamic acid 4-hydroxylase; DFR, dihydroflavonol-4-reductase; DSDG, dehydroshikimate dehydrogenase; 4CL, 4-coumarate-CoA ligase; PAL, phenylalanine ammonia lyase; STAN, *Solanum tuberosum* anthocyaninsynthase; UDP glucose, 3-*O*-flavonoid glucosyltransferase.

Anthocyanidin biosynthesis is one of the most studied plant secondary metabolite pathways. There are many genes responsible for enzyme synthesis. For almost every biosynthetic step the corresponding enzymes have been isolated and a considerable body of knowledge is available on the mechanisms that regulate their expression in the plant cell (Deroles 2009). Two classes of genes are required for anthocyanidin biosynthesis – the structural genes, for example, *ans* and *chi* that code for the enzymes that are directly involved in the production of anthocyanidin and the regulatory genes, for example, *stan1* and *stan2*, that control the transcription of structural genes (Al-Sane et al. 2011). The pathway for the anthocyanidin biosynthesis is shown in Figure 1.

In the current study, the chemical composition in terms of classes such as total antioxidant activity (TAC), total phenolic compounds (TPH) was characterized, and individual polyphenolic compounds were identified and quantified by high-performance liquid chromatography (HPLC). Molecular analysis of genes involved in the biosynthesis was performed using selected markers for *ans*, *stan1*, and *chi* genes.

This work is focused on comparing the chemical composition with botanical classification based on morphological characteristic and the similarities between the involved genes. Our hypothesis was that there is an association between the similarities, the involved genes, and the concentration patterns of anthocyanidins and antioxidants.

## Material and Methods

### Collection of potatoes

**Figure 2:**
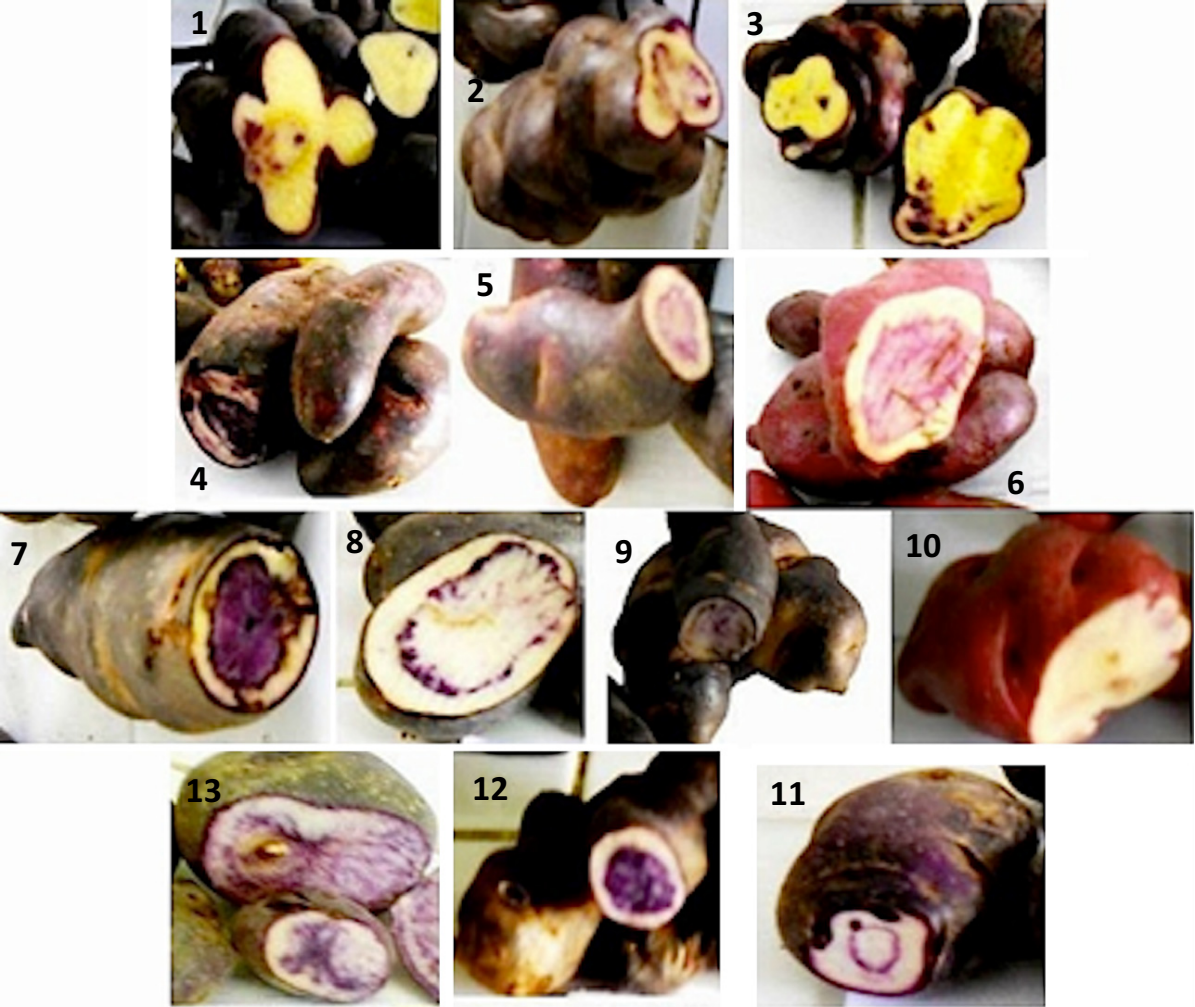
Colored potatoes collected in Bolivian Altiplano. Samples 1, 2, and 3 belong to “Ñojchajachaya” cultivar, samples from 4 to 10 belong to “Caty” cultivar, 11 belongs to “Chocapito-morado” cultivar, 12 belongs to “Ajanwiri (clon)” cultivar, and 13 belongs to “Durazno Imilla 30 CN-010” cultivar.

Thirteen purple potatoes belonging to three species (*Solanum curtilobum*, *S. ajanhuiri*, and *S. tuberosum* subsp. *andigenum*) (Fig. 2) were harvested in April 2011 during one day at Quipaquipani Research and Training Center (PROINPA Foundation). The station is located 4 km south of the town of Viacha (coordinates 68°17′49″, 16°40′17″), Ingavi Province in the Department of La Paz 3870 m.a.s.l. Each sample was taken from separate clones grown at a section of a field. The sample was obtained through a random collection of potatoes from the growing section until 1 kg was obtained. The samples were stored in plastic bags at 5°C for 6 h and then transferred to the laboratory.

### Extraction (TAC and TPH)

The potatoes were quickly washed and dried, then cut into small cubes, and about 3 g was weighed and extracted with 27 mL of methanol:water (9:1, by volume) by vortexing followed by sonication of the sample in an ice-water bath (0°C, 15 min). The mixture was centrifuged in a Thermo IEC Multi/RF with an 8850 rotor (Thermo Fisher Scientific Inc., Waltham, MA) at 20,000*g* for 30 min at 4°C, and the aspirated supernatant was stored at −80°C. The supernatants were evaluated by the ABTS (2,2′-azino-*bis*[3-ethylbenzothiazoline-6-sulfonic acid]) and TPH methods.

### Extraction (HPLC)

The potatoes were quickly washed and dried, then cut into small cubes, and about 50 g was weighed, frozen at −20°C, and lyophilized for 3 days. The dry structure was grounded and kept ready for ethanol/water extraction. One gram of each lyophilized sample was extracted with 10 mL of methanol:water (9:1, by volume) by vortexing, followed by sonicating the sample in an ice-water bath (0°C, 15 min). The mixture was centrifuged at 20,000*g* for 30 min at 4°C, and the aspirated supernatant was stored at −80°C. The supernatants were hydrolyzed and evaluated by HPLC.

### TAC evaluated by the ABTS and TPH methods

Folin-Ciocalteu reagent, gallic acid, sodium carbonate, and acetone were purchased from Merck (Darmstadt, Germany). ABTS, potassium persulfate, Trolox (6-hydroxy-2,5,7,8-tetramethyl-chroman-2-carboxylic acid, 97%), and TPTZ (2,4,6-tripyridyl-s-triazine) were obtained from Sigma-Aldrich (St. Louis, MO), ferric chloride from ICN Biomedicals (Costa Mesa, CA), glacial acetic acid and sodium acetate from BDH Chemicals (Poole, U.K.) and methanol from Laboratory Supplies (Poole, U.K.). All chemicals were of analytical grade. To oxidize the colorless ABTS to the blue–green ABTS^+^ radical cation, 5 mL of ABTS solution (7 mmol/L) was mixed with 88 μL of K_2_S_2_O_8_ (140 mmol/L) and stored at room temperature in the dark overnight. On the day of the analysis, the ABTS^+^ radical cation solution was diluted with acetate buffer to reach an absorbance of 0.70 (±0.02) at 734 nm. A Trolox standard stock solution, 5 mmol/L in ethanol, was diluted with acetate buffer to concentrations of 20–200 μmol/L. The extraction follows Nilsson et al. (2005) using modifications suggested by Peñarrieta et al. (2008). Different standards or samples (100 μL) were added to 1 mL of ABTS^+^ solution, mixed for 30 sec, after which the absorbance reading was started after another 30 sec and maintained for 6 min at 734 nm and 25°C. The concentration was plotted against percent inhibition, which was used for the calculation. The results are expressed as μmol Trolox equivalents per gram of dry matter (μmol TE/g dm).

Total phenolic compounds were measured by the Folin-Ciocalteu reagent which oxidizes the phenolic compounds to phenolates at alkaline pH in a saturated solution of sodium carbonate, resulting in a blue molybdenum–tungsten complex (Singleton and Rossi 1965). However, the method has a limited specificity and a number of substances (particularly ascorbic acid and fructose) (Prior et al. 2005) can be assumed to contribute to the apparent result (Lachman et al. 2008a). Lachman et al. (2008b) analyzed the interference systematically and provided data of reaction absorbances of potentially interfering substances. Using these data and data on fructose (Mano et al. 2007), the interference can be estimated to be less than 5%.

The Folin-Ciocalteu reagent was diluted with water (1:10 by volume) prior to analysis. A gallic acid stock solution was prepared in 80% aqueous acetone (1:1 by volume), and the gallic acid standard curve was diluted with water to concentrations of 235–1180 μmol/L. From each standard solution and sample, 50 μL was mixed with 2.5 mL of Folin-Ciocalteu reagent and 2.0 mL of sodium carbonate solution. The samples were mixed and incubated at 45°C for 30 min. The absorbance was read at 765 nm after cooling at room temperature. The absorbance of each sample was compared with the values obtained from a calibration curve using gallic acid as standard. The results are expressed as μmol gallic acid equivalents per gram of dry matter (μmol GAE/g dm) (Peñarrieta et al. 2008, 2009, 2011).

### HPLC

Baicalein (98%) internal standard, ferulic acid (99%), caffeic acid (98%), chlorogenic acid (95%), gallic acid (99%), and syringaldehyde (99%) were obtained from Sigma-Aldrich. Cyanidin and pelargonidin were obtained from Extrasynthese (Genay, France), and hydrochloric acid (36.5–38.0%) was obtained from J.T. Baker (México city, México).

Before analyzing the samples in HPLC, they were hydrolyzed by refluxing 250 μL methanol:water (9:1) extracts with 250 μL 3 N HCl and 250 μL of baicalein (internal standard) for 1 h at 90 C.

Most of the polyphenolic compounds are bound to insoluble polysaccharide components and the hydrolysis is used to release polyphenols from their corresponding glycosides.

The chemical instability during extraction and analyses of anthocyanidins is a particular concern (Reyes et al. 2004). To protect the components the samples were stored frozen, lyophilized, and the HPLC was run directly after the hydrolysis (the acid condition also stabilizes the anthocyanidins) (Fuhrman 2001; Reyes et al. 2004). The HPLC analysis was made in methanol–water extracts from lyophilized samples.

The polyphenolic compounds were separated using an Agilent liquid chromatographic system comprising a vacuum degasser (G1322 A), a solvent delivery system (Quat Pump-G1311A), an auto-injector ALS (ALS-G1329A), a column oven (ColCom-G1316A), and a diode array detector (G1315B) (Agilent, Santa Clara, CA). The column was a reverse-phase Agilent eclipse plus C18 (150 × 4.6 mm) protected by a 10 mm precolumn. The flow rate was 0.8 mL/min and the injection volume was 20 μL. The mobile phase was a binary solvent system consisting of (A) 1% acetic acid/water and (B) methanol and the gradient used was 40% B at 0 min, 65% B after 5 min, 90% B after 10 min, and 40% B after 15 min until 17 min. The UV absorbance of the eluate was recorded using a multiple diode array detector (190–550 nm). Retention times and absorbance spectrum profiles were compared with standards. Pure standards were also added to the samples as control and peak splitting was used to confirm the identification (Carrasco et al. 2011; Peñarrieta et al. 2011). The samples were quantified at 310 and 530 nm using baicalein as internal standard and using pure standards for specific absorbance determination (Peñarrieta et al. 2008).

### DNA extraction

Tris acetate, tris base, EDTA, agarose, blue dye, green dye, ethidium bromide, Flexi taq polymerase, and Mix dNTPs were obtained from Promega (Madison, WI). Sodium acetate was obtained from Sigma-Aldrich GmbH (Steinheim, Germany) and isopropanol was from Merck AG (Darmstadt, Germany).

The DNeasy Plant mini kit was obtained from Qiagen GmbH (Hilden, Gemany), BigDye Terminator v3.1 Cycle Sequencing kit was obtained from AB Applied Biosystem (Foster City, CA), 100-bp DNA ladder was obtained from Solis BioDyne (Tartu, Estonia), and the markers *21ab*, *ans*, *bch6*, *chi*, *chs*, *ct203*, *gp24*, *stan1*, *ugpase*, and *zep* were obtained from Eurogentec (San Diego, CA).

DNA was isolated from 50 mg of lyophilized sample of purple potatoes. Around 50 g of fresh sample was frozen at −20°C and had been lyophilized by LabconcoFreeZone 4.5 that reach −50°C (Labconco, Kansas, MO) and grounded into powder in mortar. A DNeasy Plant Mini extraction kit (Qiagen GmbH) was then used for DNA isolation (according to the manufacturer's instructions). The DNA quality was verified by electrophoresis on 1% agarose gel and quantified using a spectrophotometer.

### Polymerase chain reaction

Polymerase chain reaction (PCR) amplification was made using 40 ng of DNA and Master Mix (Promega), with the following concentrations: 0.2 mmol/L dNTPs, 1.5 mmol/L MgCl_2_, 0.03 μ/μL Taq DNA polymerase (Promega), and 1 μmol/L for primers (forward and reverse), respectively. Thermal cycling conditions were found by BIOER thermocycler for 10 primers referred (Zhang et al. 2009) those which are on a range of 400–900 bp: *bch6*, *21ba*, *ugp-ase*, *ans*, *ct-203*, *chi*, and *gp-24* and the following conditions: 3 min denaturation at 95°C, 1 min annealing in the range of 48–57°C and 1 min, 30 sec extension at 72°C, and the number of cycles was between 30 and 33. For primers in the range 1000–1600 bp: *stan1*, *chs*, and *zep*, the conditions were 2 min denaturation at 95°C, 1 min annealing in the range 62–65°C and 1 min, 30 sec extension at 72°C, and 35 cycles. The products obtained after PCR optimization were evaluated by horizontal electrophoresis in agarose gel (2%), stained with ethidium bromide (0.1 μL/mL; Promega), and visualized with a ultraviolet light transilluminator with the addition of a molecular weight marker 100 to 3000 bp (100-bp DNA ladder; Solis Biodyne).

### Sequence analysis

Phylogenetic trees were made based on *ans* and *stan1* genes. Primers reported by Zhang et al. (2009) were used to amplify partial regions of *ans* and *stan1*. Genes and the amplicons were purified using Qiagen DNA Cleanup Systems and sequenced. Raw sequences were imported in Geneious Pro (Ver. 5.4.6, Biomatters Ltd., Auckland, New Zealand) and homology searches were done by the Basic Local Alignment Search Tool (BLAST) to identify protein-coding regions (exons). Exon sequences were imported in MEGA 5 and aligned using multiple sequence alignment – clustalw (Tamura et al. 2011). The phylogenetic trees were built with the maximum composite likelihood model and the neighbor-joining method (Tamura et al. 2011). Bootstrap analysis was conducted with 1000 replicates. The *Ans* phylogenetic tree was constructed based on the following genes: *ans10*, *ans12*, *ans7*, *ans*6, *ans4*, *ans5* (GenBank accession number: KC752436), *ans9*, and *ans8*. It was divided in three clades called B1, B2, and B3 originating from a common ancestor, the same for *stan1* phylogenetic tree, respectively. The s*tan* phylogenetic tree was constructed based on the following genes: *stan12*, *stan9*, *stan3*, *stan1*, *stan2*, *stan10* (GeneBank accession number: KC752437), *stan6*, *stan8*, *stan5*, and s*tan11*. It was divided in three clades called A1, A2, and A3 originating from a common ancestor.

### Statistical analysis

The principal component analysis (PCA) was performed using MATLAB Release 2012a (MathWorks, Natick, MA).

## Results and Discussion

### Chemical composition of different cultivars

**Figure 3:**
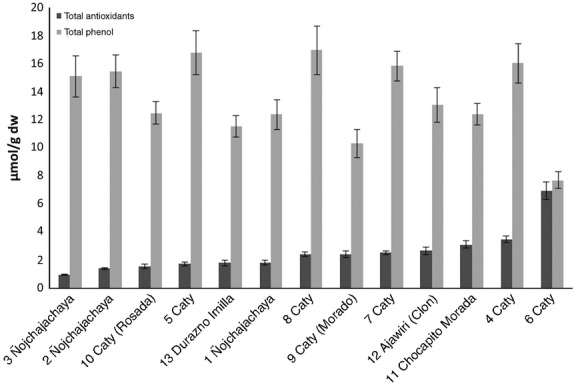
Total antioxidants expressed as trolox equivalent/g dry weight and total phenols expressed as gallic acid equivalent/g dry weight of different colored potato. Values are means of triplicate measurements ± standard error of mean.

TAC and TPH of colored potatoes are shown in Figure 3, ranging from 1.0 to 7.0 μmol Trolox/g dry weight (dw) and 8.0–17.0 μmol GAE/g dw, respectively. The TAC values obtained are similar to those reported in the literature for potato (1 μmol Trolox/g dw) (Peñarrieta et al. 2011) as shown in Table 1. The TPH results are similar to those reported previously for potatoes (10 μmol GAE/g dw) (Peñarrieta et al. 2011) as well as of purple-fleshed potatoes (19.3 μmol GAE/g dw) (Lachman et al. 2008a,2008b) in Stachy locality of Czech Republic with environmental conditions similar to that in the Andean region (lower temperature and higher altitude; see Table 1).

**Table 1:** Comparison of present study data in colored potato with literature data.

*Solanum* cultivar	TAC (μmol Trolox/g dw)	TPH (μmol GAE/g dw)
This study		
*S. stenotomum* (colored potato)	1–7	8–17
Literature data		
*S. tuberosum* (colored potato)^1^		9-35
*S. tuberosum* L. (purple-flesh potato)^2^		19
*S. tuberosum* L.^3^	1	10

1 TPH evaluated by Folin-Ciocalteu reagent (Andre et al. 2007).

2 TPH evaluated by Folin-Ciocalteu reagent made by ABTS method (Lachman et al. 2008, 2008).

3 TAC evaluated by ABTS method and TPH by Folin-Ciocalteu reagent (Peñarrieta et al. 2011).

**Figure 4:**
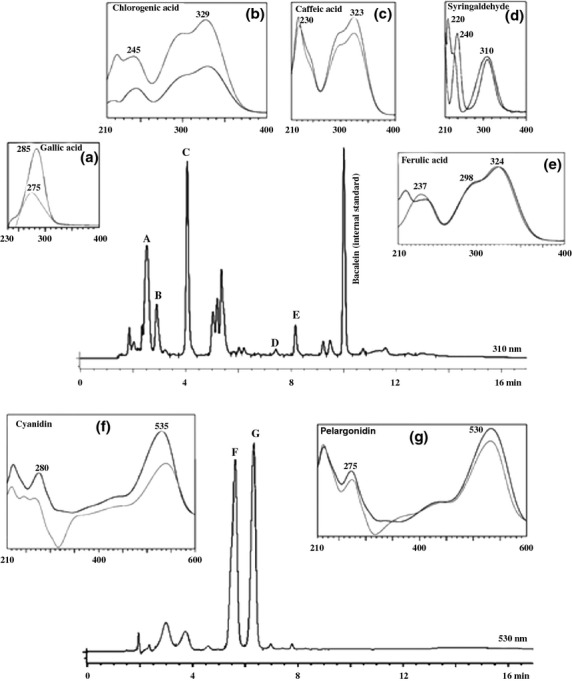
Chromatogram of polyphenolic compounds identified in colored potatoes. Black peak spectra belong to the standards and gray peaks belong to the samples.

The polyphenols were identified and quantified (Table 2) by comparison with standards using HPLC with a UV–Vis diode array detector. Seven individual compounds were identified: cyanidin, pelargonidin, ferulic acid, caffeic acid, chlorogenic acid, gallic acid, and syringaldehyde (Figure 4). Baicalein was used as the internal standard (Peñarrieta et al. 2011).

**Table 2:** Content of individual polyphenolic compounds in colored potato samples in methanol–water extracts, expressed as μmol/g dw.

Cultivar	Sample	Cyanidin	Pelargonidin	Ferrulic acid	Caffeic acid	Chlorogenic acid	Gallic acid	Syringaldehyde
Ñojchajachaya	1	0.07	0.07	0.01	0.09	0.3	1.5	0.01
Ñojchajachaya	2	0.04	0.06	0.01	0.08	0.2	1.6	0.00
Ñojchajachaya	3	0.01	0.01	0.02	0.07	0.3	4.1	0.00
Caty	4	0.01	0.005	0.02	0.08	0.5	1.5	0.00
Caty	5	0.02	0.02	0.04	0.13	0.4	7.6	0.00
Caty	6	0.003	0.03	0.01	0.06	0.2	1.2	0.00
Caty	7	0.02	0.01	0.02	0.10	0.5	3.4	0.00
Caty	8	0.01	0.03	0.02	0.07	0.2	1.8	0.00
Caty (Morado)	9	0.02	0.02	0.03	0.09	0.2	5.1	0.00
Caty (Rosada)	10	0.03	0.01	0.01	0.07	0.3	2.6	0.00
Chocapito-Morada	11	0.05	0.02	0.01	0.08	0.2	2.9	0.00
Ajawiri (Clon)	12	0.03	0.01	0.01	0.06	0.2	2.9	0.00
Durazno Imilla	13	0.01	0.004	0.01	0.03	0.04	3.7	0.00
Median		0.02	0.02	0.01	0.08	0.2	2.9	0.00
SD		0.02	0.02	0.01	0.02	0.1	1.8	0.00
SEM		0.01	0.01	0.002	0.01	0.04	0.5	0.001
Range		<0.003–0.07	<0.004–0.07	<0.01–0.04	<0.03–0.13	<0.04–0.5	<1.2–7.6	<0.0–0.01

The absorption maxima for cyanidin and pelargonidin were found at 530 nm and for ferulic acid, caffeic acid, chlorogenic acid, gallic acid, and syringaldehyde at 310 nm. The presence of pelargonidin and cyaniding has been reported in Shetland Black cultivar purple-flesh potato by Lachman et al. (2012). Also, the polyphenols identified in this study have been reported before in purple-fleshed potatoes (Friedman 1997; Lewis et al. 1998; Del Mar Verde Méndez et al. 2004).

The quantitative data are summarized in Table 2. The amount of individual polyphenolic compound varies between cultivars. Gallic acid is the main polyphenolic compound in all samples followed by chlorogenic acid and caffeic acid, while ferulic acid is present at lower concentrations. The caffeic acid concentration is similar among the samples, while cyanidin and pelargonidin levels are higher in some of the “Ñojchajachaya” samples (Table 2).

Pelargonidin values obtained (0.004–0.07 μmol/g dw) were comparable to that found in the Shetland Black cultivar purple-flesh potato (0.02 μmol/g dw) (Lachman et al. 2012), but the results were lower than those reported in the literature for red potato (2.3–23.3 μmol/g dw) (Lewis et al. 1998). Also, cyanidin values (0.003–0.07 μmol/g dw) were comparable to that found in Shetland Black cultivar (0.01 μmol/g dw), which was the only purple-flesh potato with both anthocyanidins reported (Lachman et al. 2012).

The ferulic acid content (0.01–0.04 μmol/g dw) is in agreement with literature values for pink potato (0.01 μmol/g dw) (Del Mar Verde Méndez et al. 2004). The caffeic acid values (0.03–0.1 μmol/g dw) are also in agreement with literature data obtained from pink potato (0.1 μmol/g dw) (Del Mar Verde Méndez et al. 2004). On the other hand, lower values were obtained for chlorogenic acid content in this study (0.04–0.5 μmol/g dw) compared with literature data (1 μmol/g dw) (Del Mar Verde Méndez et al. 2004; Peñarrieta et al. 2011).

The values for gallic acid (1.2–7.6 μmol/g dw) are slightly higher than previously reported data (1.1 μmol/g dw). The values for syringaldehyde (0.01 μmol/g dw) are similar to values reported previously (Peñarrieta et al. 2011).

**Figure 5:**
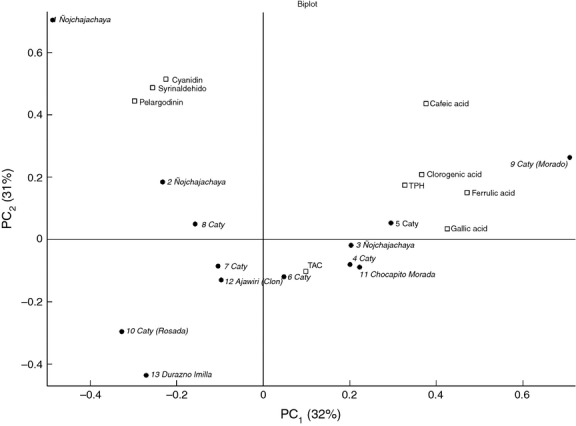
PCA analysis (biplot) of anthocyanin, phenolic compounds, total phenol, and total antioxidant content in 13 different purple potatoes.

PCA was used to reveal potential patterns between the 13 colored potato samples and TAC, TPH, concentrations of individual anthocyanidins and polyphenolic compounds are the nine measured parameters. The explained total variance of PC1 is 32% and of PC2 is 32% (Fig. 5).

The “Nojchajachaya” group is scattered along the second PC, with some samples displaying a high concentration of cyanidin, pelargonidin, and syringaldehyde, whereas one sample displays low concentrations. These samples also have similar levels of TAC (Fig. 3). The Caty group is scattered around the center of the coordinates and therefore does not display any pattern. The results show that the botanical classification only describes limited aspects of the variability of this material.

### Analysis of genes associated with the biosynthesis of anthocyanidin

**Figure 6:**
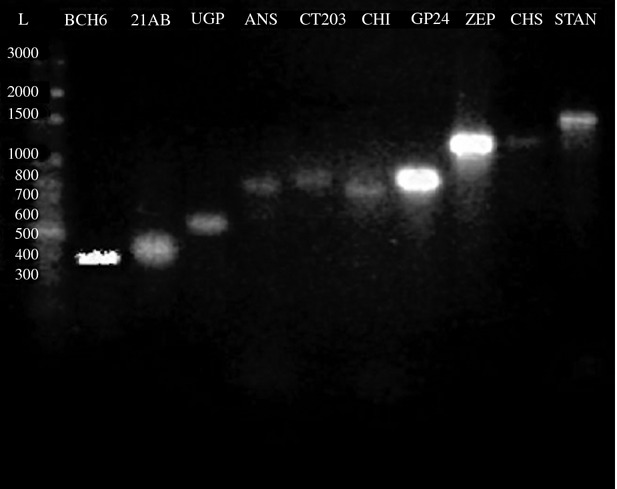
Genomic DNA of Durazno Imilla 30CN-010 sample was amplified by *Bch6*, *21BA*, *UGPasa*, *CT-203*, *Chi*, *GP-24*, *Stan1*, *Chs*, and *Zep* primers, restricted with Taq DNA polymerase, and electrophoresed through a 2% agarose gel. The primers are listed in ascending order in a range of 400–1600 bp.

In the beginning about 10 genes associated with anthocyanidin were analyzed (Fig. 6). However, only three (*chi*, *ans*, and *stan1*) of the 10 genes are directly associated with the biosynthesis of anthocyanidin in colored potato. The three genes, *chi*, *ans*, and *stan1*, were present in all individual samples as confirmed by gel electrophoresis (Fig. 6). The three genes of each sample were sequenced. The size of the fragments (500 bp) of the *chi* gene material was too small to allow for a proper phylogenetic analysis. The phylogenetic trees were made for *ans* (800 bp) and *stan1* (1200 bp) sequences. For some samples, individual samples had to be excluded due to poor resolution; for the *ans* gene, only eight could be used and for the *stan1* gene only 10 could be used of the 13 individual samples.

**Figure 7:**
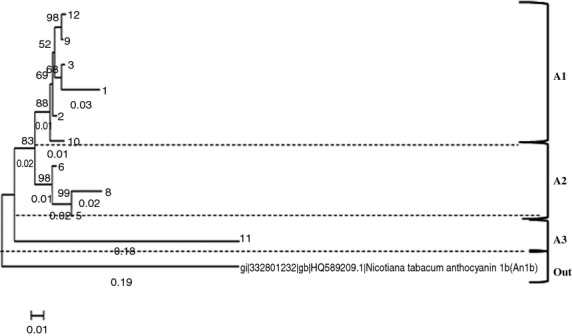
*Stan* gene phylogenetic tree constructed by coding exon partial area with approximately 300–500 base pairs made by the Neighbor-Joining algorithm.

The *stan1*, from here on only termed *stan*, phylogenetic tree clearly shows how samples of the *stan* gene are grouped together, how they originate from the same node, and that they are completely separated from *Nicotiana tabacum,* thereby confirming the uniformity in the material making contamination unlikely (Fig. 7).

The s*tan* phylogenetic tree is divided into three clades by common nodes. The genes *stan12* (i.e., the *stan* gene from sample 12), *stan9*, *stan3*, *stan1*, *stan2*, and *stan10* are grouped in the same clade called A1 and they have 88% similarity. In clade A2 the genes *stan6*, *stan8*, and *stan5* are branched together with a common node with a similarity above 98%. Clade A3 (s*tan11*) shows 83% similarity with clades A1 and A2.

**Figure 8:**
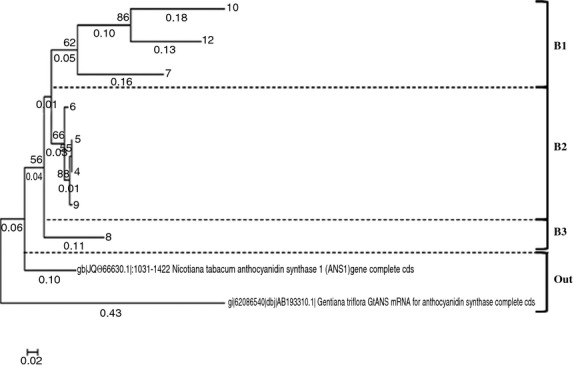
*Ans* gene phylogenetic tree constructed by coding exon partial area with approximately 300–500 base pairs made by the Neighbor-Joining algorithm.

The *ans* phylogenetic tree can be divided into three clades (B1, B2, and B3) that are grouped together and are well separated from *N. tabacum* and *Gentiana triflora* (Fig. 8). In clade B1 there are genes from thee samples *ans10*, *ans12*, and *ans7*, which belong to a common node with a 62% of similarity. Clade B2 is formed by *ans*6, *ans4*, *ans5*, and *ans9*, with a common node, and clade B3 is formed by *ans8* with 55% similarity to clades B1 and B2.

The explanatory power of the analysis of the *ans* genes of samples may reflect the larger biological variation in the *ans* gene compared to the *stan* gene.

The botanical classification is based on morphological characteristics of the tubers (Table 3), and the *stan* and *ans* phylogenetic trees (Figs. 7, 8) show no direct similarity as the different cultivars are not grouped in the phylogenetic trees.

**Table 3:** Description of potatoes (*Solanum* sp.) collected in La Paz, Bolivia.

Code	Cultivar	Scientific name	Dry matter (%)
1	Ñojchajachaya	*S. tuberosum* subsp. *andigenum*	33
2	Ñojchajachaya	*S. tuberosum* subsp. *andigenum*	26
3	Ñojchajachaya	*S. tuberosum* subsp. *andigenum*	29
4	Caty	*S. tuberosum* subsp. *andigenum*	31
5	Caty	*S. tuberosum* subsp. *andigenum*	28
6	Caty	*S. tuberosum* subsp. *andigenum*	24
7	Caty	*S. tuberosum* subsp. *andigenum*	27
8	Caty	*S. tuberosum* subsp. *andigenum*	33
9	Caty (Morado)	*S. tuberosum* subsp. *andigenum*	28
10	Caty (Rosada)	*S. tuberosum* subsp. *andigenum*	31
11	Chocapito-Morada	*S. curtilobum*	25
12	Ajawiri (Clon)	*S. ajanhuiri*	26
13	Durazno Imilla 30CN-010	*S. tuberosum* subsp. *andigenum*	32

### Correlation between phylogenetic trees and chemical composition

**Figure 9:**
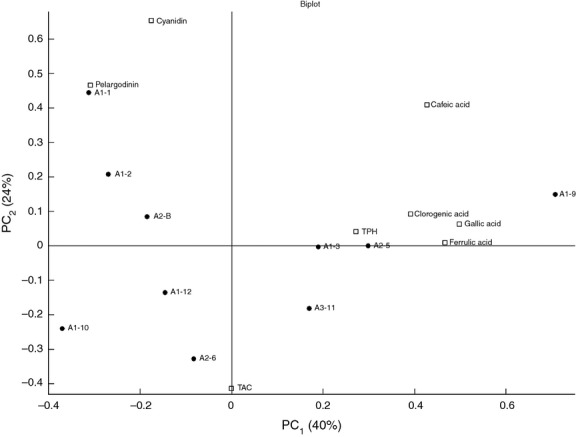
PCA analysis (biplot) of chemical composition in 10 different purple potatoes and clades belonging to the *stan* phylogenetic tree.

PCA was used to reveal patterns among clades from *stan* and *ans* phylogenetic trees and the chemical composition of individual samples. The PCA of the eight *stan* samples gave PC1 and PC2 together explaining 64% of the total variance (Fig. 9). The three clades A1, A2, and A3 enclose each other. Thus, this result shows that the variance in the chemical composition is not explained by differences in the *stan* gene.

**Figure 10:**
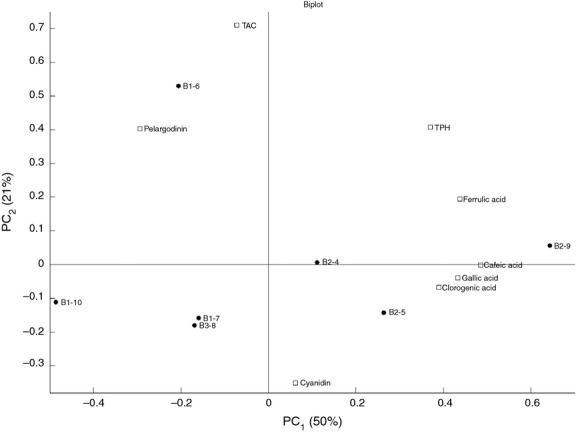
PCA analysis (biplot) of chemical composition in seven different purple potatoes and clades belonging to the *ans* phylogenetic tree.

Figure 10 shows the corresponding PCA for the *ans* gene for the eight samples where PC1 and PC2 together explain 71% of the variance. Clades B1 and B2 are from two separated areas, indicating that the *ans* gene has a clear impact on the chemical composition. Clade B1 has a correlation with a somewhat higher level of pelargonidin. B2 is located along the positive PC1 and thereby correlates with high levels of ferulic acid, gallic acid, caffeic acid, chlorogenic acid, and TPH (Fig. 10).

The *ans* gene is directly involved in anthocyanidin synthesis. The anthocyanidin has a more final position in the biochemical pathways (Singleton and Rossi 1965; Nilsson et al. 2005; Mano et al. 2007; Peñarrieta et al. 2008; Davies et al. 2009).

The *stan* gene controls dihydroxyflavonol-4-reductase activity (Bai et al. 2011; Lachman et al. 2012) that leads to the formation of leucoanthocyanins that may be converted into anthocyanidins or into cathechins and condensed lignins (Fig. 1). Thus a less clear connection can be assumed. Thus potato color is confirmed as a sign not only of high level of anthocyanidins but also of polyphenols in general (Fuhrman 2001).

Naturally, not only the genetics are expected to determine the chemical composition as other aspects – *extrinsic factors*: environmental conditions, cooler temperatures (Reyes et al. 2004) growing conditions (Lachman et al. 2008a; Hamouz et al. 2010) – and the *intrinsic factor*: the cultivar (Andre et al. 2007; Hamouz et al. 2010) – may play a role. However, the samples in this article were collected at an experimental farm with uniform growing conditions, which should limit the influence of extrinsic factors on the results.

## Conclusion

The results of this study show the existence of a limited correlation between botanical classification, based on morphological identification, and polyphenol composition. No correlation between genetic grouping of the *ans* and *stan* genes and botanical classification was found. However, it was possible to identify a correlation between the *ans* gene clades and the levels of anthocyanidins as well as of other polyphenols. Thus, this result confirms the concept that potato color can be used in the search for high polyphenol potato cultivars.

## References

[b1] Alcalde-Eon C, Saavedra G, Pascual-Teresa S, Rivas-Gomzalo J (2004). Identification of anthocyanins of pinta boca (*Solanum stenotomum*) tubers. Food Chem.

[b2] Al-Sane KO, Povero G, Perata P (2011). Anthocyanin tomato mutants: overview and characterization of an anthocyanin-less somaclonal mutant. Plant Biosyst.

[b3] Andre C, Ghislain M, Bertin P, Oufir M, Herrera MR, Hoffman L (2007). Andean potato cultivars (*Solanum tuberosum* L.) as a source of antioxidant and mineral micronutrients. J. Agric. Food Chem.

[b4] Bai Y, Pattanaik S, Patra B, Werkman J, Xie C, Yuan L (2011). Flavonoid-related basic helix-loop-helix regulators, NtAn1a and NtAn1b, of tobacco have originated from two ancestors and are functionally active. Planta.

[b5] Brown C (2005). Antioxidant in potato. Am. J. Potato Res.

[b6] Carrasco C, Baudel H, Peñarrieta M, Solano C, Tejeda L, Roslander C (2011). Steam pretreatment and fermentation of the straw material “Paja Brava” using simultaneous saccharification and co-fermentation. J. Biosci. Bioeng.

[b7] Davies KM, Zhang H, Schwinn KE, Daayf F, Lattanzio V (2009). Recent advances in polyphenol research. Recent advances in the molecular biology and metabolic engineering of flavonoid biosynthesis in ornamental plants.

[b8] Del Mar Verde Méndez C, Rodríguez Delgado MÁ, Rodríguez Rodríguez EM, Díaz Romero C (2004). Content of free phenolic compounds in cultivars of potatoes harvested in Tenerife (Canary Islands). J. Agric. Food Chem.

[b9] Deroles S, Winefield C, Davies K, Gould K (2009). Anthocyanin biosynthesis in plant cell cultures: a potential source of natural colorants. Anthocyanins: biosynthesis, functions, and applications.

[b10] Friedman M (1997). Chemistry, biochemistry, and dietary role of potato polyphenols. Agric. Food Chem.

[b11] Fuhrman B, Aviram M, Cadenas E, Packer L (2001). Polyphenols and flavonoids protect LDL against atherogenic modifications. Handbook of antioxidants.

[b12] Hamouz K, Lachman J, Hejtmankova A, Pazderu K, Cizek M, Dvorak P (2010). Effect of natural and growing conditions on the content of phenolics in potatoes with different flesh colour. Plant Soil Environ.

[b13] Hamouz K, Lachman J, Pazderu K, Tomasek J, Hejtmankova K, Pivec V (2011). Differences in anthocyanin content and antioxidant activity of potato tubers with different flesh colour. Plant Soil Environ.

[b14] Lachman J, Šulc M, Orsák M, Hamouz K, Dvořák P (2008a). Differences in phenolic content and antioxidant activity in yellow and purple-fleshed potatoes grown in the Czech Republic. Plant Soil Environ.

[b15] Lachman J, Hamouz K, Orsak M, Pivec V, Dvorak P (2008b). The influence of flesh colour and growing locality on polyphenolic content and antioxidant activity in potatoes. Sci. Hort. Amsterdam.

[b16] Lachman J, Hamouz K, Orsak M, Pivec V, Hejtmankova K, Pazderu K (2012). Impact of selected factors – cultivar, storage, cooking and baking on the content of anthocyanins in coloured-flesh potatoes. Food Chem.

[b17] Lewis C, Walker J, Lancaster J, Sutton K (1998). Determination of anthocyanins, flavonoids and phenolic acids in potatoes. I: Colored cultivars of *Solanum tuberosum* L. Sci. Food Agric.

[b18] Mano H, Ogasawara F, Sato K, Higo H, Minobe Y (2007). Isolation of regulatory gene of anthocyanin biosynthesis in tuberous roots of purple-fleshed sweet potato. Plant Physiol.

[b19] Nilsson J, Pillai D, Önning G, Persson C, Nilsson Å, Åkesson B (2005). Comparison of the 2,2′-azinobis-3-ethylbenzothiazoline-6-sulfonic acid (ABTS) and ferric reducing anti-oxidant power (FRAP) methods to assess the total antioxidant capacity in extracts of fruit and vegetables. Mol. Nutr. Food Res.

[b20] Peñarrieta J, Alvarado J, Åkesson B, Bergenståhl B (2008). Total antioxidant capacity and content of flavonoids and other phenolic compounds in canihua (*Chenopodium pallidicaule*): an Andean pseudocereal. Mol. Nutr. Food Res.

[b21] Peñarrieta J, Alvarado J, Bergenståhl B, Akesson B (2009). Total antioxidant capacity and content of phenolic compounds in wild strawberries (*Fragaria vesca*) collected in Bolivia. Int. J. Fruit Sci.

[b22] Peñarrieta J, Salluca T, Tejeda L, Alvarado J, Bergenståhl B (2011). Changes in phenolic antioxidants during chuño production (traditional Andean freeze and sun-dried potato). J. Food Compos. Anal.

[b23] Preedy VR, Watson RR, Patel V (2011). Nuts and seeds in health and disease prevention.

[b24] Prior R, Wu X, Schaich K (2005). Standardized methods for the determination of antioxidant capacity and phenolics in foods and dietary supplements. J. Agric. Food Chem.

[b25] Reyes LF, Miller JC, Cisneros-Zevallos L (2004). Environmental conditions influence the content and yield of anthocyanins and total phenolics in purple- and red-flesh potatoes during tuber development. Am. J. Potato Res.

[b26] Singleton V, Rossi J (1965). Colorimetry of total phenolics with phosphomolybdic-phosphotungstic acid reagent. Am. J. Enol. Vitic.

[b27] Stushnoff C, Ducreux LJM, Hancock RD, Hedley PE, Holm DG, McDougall GJ (2010). Flavonoid profiling and transcriptome analysis reveals new gene–metabolite correlations in tubers of *Solanum tuberosum* L. J. Exp. Bot.

[b28] Tamura K, Peterson D, Peterson N, Stecher G, Nei M, Kamur S (2011). MEGA5: molecular evolutionary genetics analysis using maximum likelihood, evolutionary distance, and maximum parsimony methods. Mol. Biol. Evol.

[b29] Zhang Y, Jung C, De Jong W (2009). Genetic analysis of pigmented tuber flesh in potato. Theor. Appl. Genet.

